# An overview on the treatments and prevention against COVID-19

**DOI:** 10.1186/s12985-023-01973-9

**Published:** 2023-02-08

**Authors:** Yunes Panahi, Armita Mahdavi Gorabi, Sona Talaei, Fatemeh Beiraghdar, Abolfazl Akbarzadeh, Vahideh Tarhriz, Hassan Mellatyar

**Affiliations:** 1 Pharmacotherapy Department, Faculty of Pharmacy, Bagyattallah University of Medical Sciences, Tehran, Iran; 2 Chronic Diseases Research Center, Endocrinology and Metabolism Population Sciences Institute, Tehran University of Medical Sciences, Tehran, Iran; 3 Department of Basic Sciences, Maragheh University of Medical Sciences, Maragheh, Iran; 4 Nephrology and Urology Research Center, Baqiyatallah University of Medical Sciences, Tehran, Iran; 5 Department of Medical Nanotechnology, Faculty of Advanced Medical Sciences, Tabriz University of Medical Sciences, Tabriz, Iran; 6 Infectious and Tropical Diseases Research Center, Tabriz University of Medical Sciences, Tabriz, Iran

**Keywords:** Coronaviruses, COVID-19, SARS-CoV-2, Antiviral agents, Biologic agents, Anti-inflammatory agents, Herbal agents, Vaccine

## Abstract

**Background:**

The coronavirus disease 2019 (COVID-19) caused by the severe acute respiratory syndrome coronavirus 2 (SARS-CoV-2) continues to plague the world. While COVID-19 is asymptomatic in most individuals, it can cause symptoms like pneumonia, ARDS (acute respiratory distress syndrome), and death in others. Although humans are currently being vaccinated with several COVID-19 candidate vaccines in many countries, however, the world still is relying on hygiene measures, social distancing, and approved drugs.

**Result:**

There are many potential therapeutic agents to pharmacologically fight COVID-19: antiviral molecules, recombinant soluble angiotensin-converting enzyme 2 (ACE2), monoclonal antibodies, vaccines, corticosteroids, interferon therapies, and herbal agents. By an understanding of the SARS-CoV-2 structure and its infection mechanisms, several vaccine candidates are under development and some are currently in various phases of clinical trials.

**Conclusion:**

This review describes potential therapeutic agents, including antiviral agents, biologic agents, anti-inflammatory agents, and herbal agents in the treatment of COVID-19 patients. In addition to reviewing the vaccine candidates that entered phases 4, 3, and 2/3 clinical trials, this review also discusses the various platforms that are used to develop the vaccine COVID-19.

## Introduction

There is a current outbreak of a novel coronavirus, the severe acute respiratory syndrome coronavirus 2 (SARS-CoV-2). SARS-CoV-2 is an etiologic agent of coronavirus disease 2019 (COVID-19), which appeared in Wuhan, China, and now spread worldwide [1, 2]. Compared to other members of the Coronaviridae family, such as the SARS-CoV and MERS-CoV (Middle East respiratory syndrome coronavirus), the mortality rate of SARS-CoV-2 is considerably lower; however, it is more transmissible. So far, > 210 million infected cases and > 4.5 million deaths from SARS-CoV-2 infection have been identified. The coronavirus is transmitted commonly by respiratory droplets and can be asymptomatic between 2 and 14 days. This oscillating period makes it notably hard to reach a primary diagnosis and initiate treatment on time. The most frequent signs of COVID-19 are cough, muscle pain, fever, shortness of breathing, sore throat, fatigue, headache, a loss of smell and taste, and dyspnea [3]. The main target of SARS-CoV-2 in patients is the lower respiratory tract. It is noticeable that adults mostly present with a significant reduction in CD8^+^ and CD4^+^ T-cells in the primary stages of the disease [4]. Following this decrease, patients suffer from ARDS for approximately 7–10 days after the beginning of the disease because of fast viral replication, the release of a storm of pro-inflammatory cytokines, initiating chemokine responses, and infiltration of inflammatory cells [5]. In addition to ARDS, complications like neurological complications, RNAaemia, and multi-organ failure are reported in these patients [6]. The progression of severe COVID-19 is associated with possible risk factors such as old-aged, diabetic, hypertensive, immunocompromised patients, and patients with primary diseases such as cancer and neurodegenerative diseases [2, 7, 8].

**Fig. 1:**
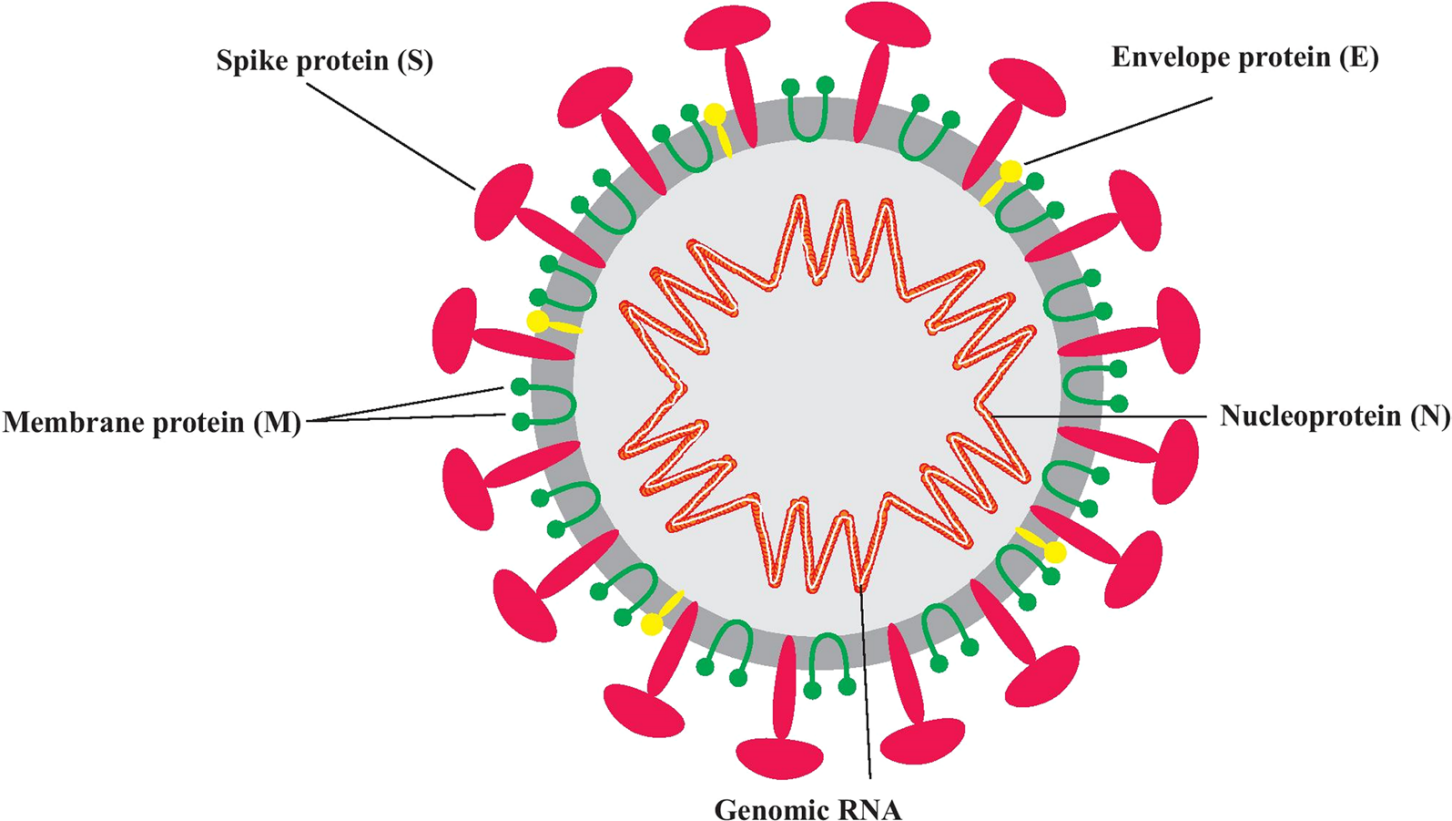
Structure of SARS-CoV-2

**Fig. 2:**
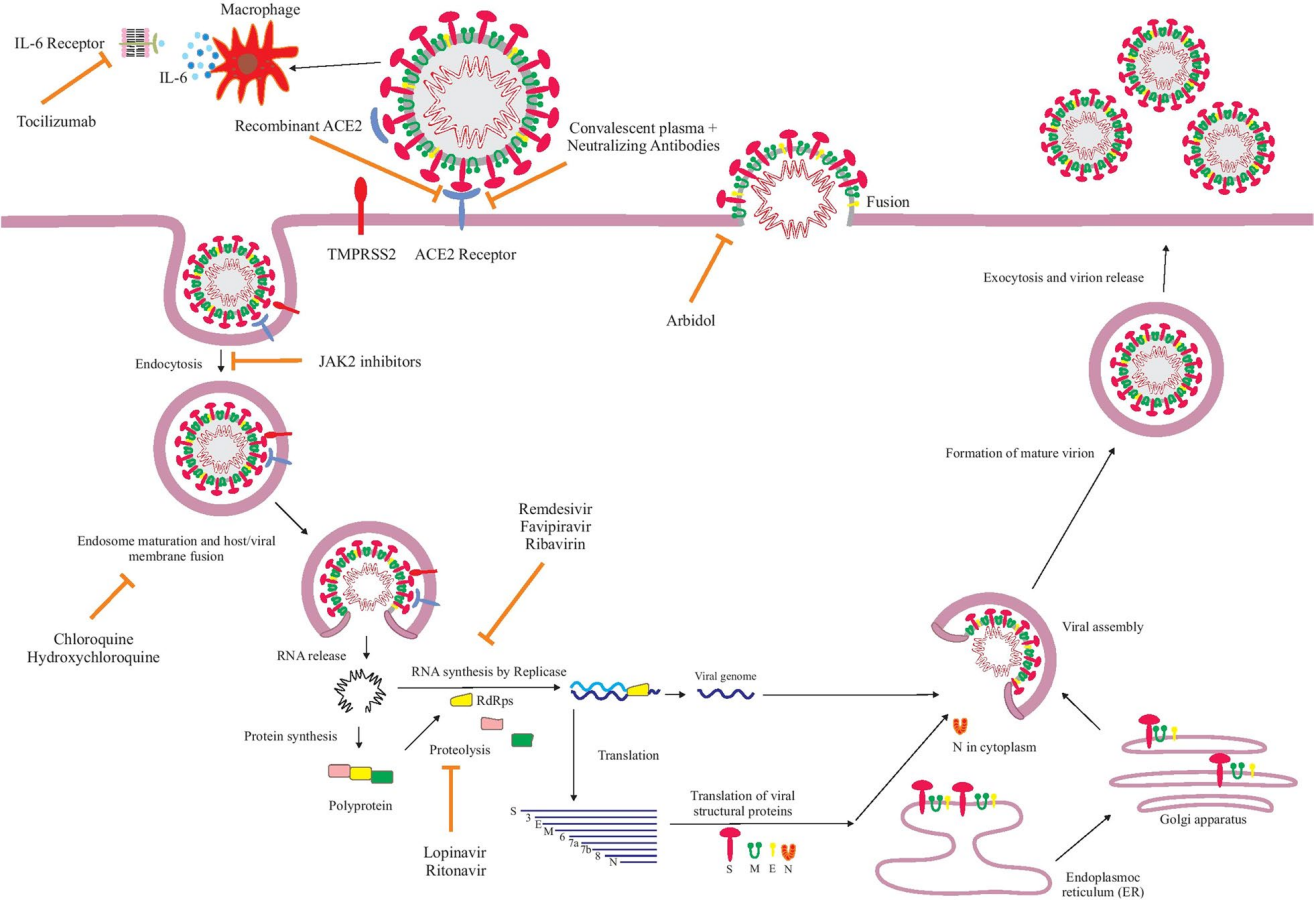
The replication cycle of SARS-CoV-2 with target therapeutic agents

SARS-CoV-2 is a single-stranded RNA beta-coronavirus with positive polarity and helical symmetry of the nucleocapsid. The structure of the SARS-CoV-2 contains four various structural proteins: spike (S), nucleocapsid (N), membrane (M), and envelope (E) (Fig. 1) [9, 10]. Coronaviruses possess the largest genome among RNA viruses (~ 30-kb nucleotides) and contain at least six open reading frames (ORFs). The first ORF encodes a replicase protein and other ORFs encode structural proteins [11]. The spike proteins are directed outwards from the membrane, and two envelope and membrane proteins are placed among the spike proteins. The role of the nucleocapsid proteins is to condense the RNA genome, which seizes the cell protein machinery in the replication cycle of the virus. The S-protein, containing subunits of S1 and S2, has an important function in binding between virus and ACE2 receptor [12]. S1 attaches to ACE2 receptor of the host cell and S2 is activated through host transmembrane protease serine subfamily member 2 (TMPRSS2) that drives membrane fusion [13]. After entering into the cell, SARS-CoV-2 utilizes endogenous cellular machinery of the host for the transcription, replication, and translation of its RNA genome to various viral proteins which are required for reassembling, encapsulating, and exocytosis of newly created virions from the cell (Fig. 2) [14]. Regrettably, there is no standard prevention and treatment against COVID-19 presently. With an understanding of SARS-CoV-2 structure, its infection mechanisms, and the clinical signs of patients, several treatments had been utilized for COVID-19 clinical studies. So far, therapeutic options for COVID-19 can be classified as antiviral agents, biologic agents, anti-inflammatory agents, and herbal agents. Besides the discovered therapeutic agents, vaccines are also in preclinical and clinical trials. This review focuses on the potential treatments that have been employed up to now in treatment of COVID-19 disease.

## Therapeutic agents

### Antiviral agents

Antiviral agents are small molecules that act as inhibitors of different steps of the virus life cycle (Fig. 2). In the primary transmission period of SARS-CoV-2, no antiviral agent has been proven to be efficient for COVID-19 disease. With the accumulation of clinical experiences and the deepening of research, types of antiviral agents are considered potential drugs for the infection of COVID-19.

#### Remdesivir

Remdesivir is found to be the most promising antiviral agent among different potential agents tested for the treatment of the disease COVID-19. At first, Remdesivir was developed for the treatment of hepatitis C, and was subsequently repurposed as a therapeutic agent against Ebola and Marburg virus infections before being considered as a treatment for COVID-19. Remdesivir is an adenosine analog, which enters the host cell in the form of a monophosphoramidate prodrug, then metabolized to an analog of adenosine triphosphate. Remdesivir acts by targeting viral RNA-dependent RNA polymerase (RdRp) and prevents the replication of the virus by premature termination of RNA transcription. Remdesivir possesses wide antiviral activity on many virus families such as paramyxoviruses, pneumoviruses, filoviruses, and coronaviruses (e.g., MERS-CoV and SARS-CoV) [15]. M. Wang and colleagues in an in vitro trial showed that Remdesivir potently prevented SARS-CoV-2 with an EC_50_ value of 1.76 μMol in Vero E6 cells [16]. Treatment with Remdesivir indicated clinical improvement for the first COVID-19 patient in the United States [17] and then trials have been started to assess its efficacy in hospitalized COVID-19 patients. In a recent study, clinical recovery was seen in 68% of severe COVID-19 patients who were treated with Remdesivir [18]. Several meta-analyses have been conducted to assess the antiviral activities of Remdesivir on COVID-19 patients. Based on obtained results, Remdesivir did not any significant difference in mortality rate in hospitalized adults with COVID-19; however, it can improve the percent recovered, decreases serious injuries, and probably leads to a decline in the rate of mechanical ventilation [19–21]. The National Institutes of Health guidelines later recommended that combining Remdesivir with anti-inflammatory drugs such as Tocilizumab, corticosteroids, and Baricitinib, can increase the benefit observed across all endpoints in patients with pneumonia and on oxygen support [22–24]. Recently have been indicated that early Remdesivir therapy in patients with active malignancies reduces 28-day in-hospital mortality by 80% [25].

#### Favipiravir

Favipiravir is a guanine analog that indicated antiviral activity against new or re-emerging influenza, Ebola, and yellow fever. Favipiravir is intracellularly phosphoribosylated to favipiravir-ribofuranosyl-5ʹ-triphosphate, which can function as a substrate of RdRp of RNA viruses [26]. It is found that Favipiravir prevented the SARS-CoV-2 infection with an EC_50_ of 61.88 μMol in Vero E6 cells [16]. An open-label control trial to investigate the clinical effect of Favipiravir in COVID-19 patients was performed. The results indicated that the Favipiravir arm had a shorter viral clearance median time than the control arm [4 days versus 11] and also had an improvement rate of 91.43% versus 62.22% in chest imaging [27]. A randomized clinical study indicated that Favipiravir had a clinical improvement rate of day 7 and resulted in improved latency to relief for cough and pyrexia than Arbidol [28].

#### Ribavirin

Ribavirin is a guanosine analog, which possesses antiviral activity on many RNA viruses, including respiratory syncytial virus, hepatitis C virus, and virus hemorrhagic fevers. Ribavirin exerts its antiviral activity through multimodal mechanisms. The antiviral mechanisms of Ribavirin include interference with polymerase, inhibition of nucleotide biosynthesis, interference with RNA capping, and fatal mutagenesis [29]. Ribavirin was selected for COVID-19 patients in combination with Interferon or Ritonavir/Lopinavir in accordance with clinical guidelines in China (http://www.nhc.gov.cn/xcs/zhengcwj/202002/a5d6f7b8c48c451c87dba14889b30147.shtml). It is found that Ribavirin cannot decrease viral RNA when given alone, therefore, it is given in combination with Interferon-α to modulate and increase host immunity [30]. In an in vitro study, was found that a high dose of Ribavirin is needed to reduce viral infection of SARS-CoV-2 [16]. However, Ribavirin possesses a related risk of hemolytic anemia and remarkable hemoglobin decrease, which is dangerous for patients in respiratory distress [31]. In a retrospective cohort trial is indicated that Ribavirin therapy in severe COVID-19 patients isn't related to reduced negative conversion time for SARS-CoV-2 test and also a reduced mortality rate [32].

#### Interferons (INFs)

IFNs, known as cytokines, activate innate immune response after the virus infection. IFNs-α/β are wide-range antiviral agents that, in addition to activating the innate immune system, inhibit virus replication by interaction with toll-like receptors. IFN-α has no antiviral effect directly, while IFN-β can stimulate protein synthesis with immunomodulatory and antiviral activities. IFN-α/β both indicated antiviral activity against MERS-CoV and SARS-CoV in vitro [33]. Although IFN-α is found to be effective against SARS-CoV, its selectivity index is low than IFN-β. A retrospective cohort study indicated that a combination of INF-α with Ribavirin improved the clinical situation of patients with MERS-CoV [31]. A triple-combination study of IFN-β1b, Ribavirin, and Lopinavir/Ritonavir demonstrated that significant differences were seen in symptoms relief, shorten the viral shedding duration, and hospital stay in the combination group [34]. A recent exploratory study indicated that IFN-α2b alone or in combination with Arbidol decreased the SARS-CoV-2 duration in the upper respiratory tract and parallel decreased the blood levels of inflammatory markers like interleukin-6 and C-reactive protein [35].

#### Ritonavir/Lopinavir

Ritonavir/Lopinavir are protease inhibitors utilized to treat HIV infection. Proteases are vital enzymes in coronavirus's polyprotein processing. Lopinavir as the HIV protease inhibitor can weaken the virus infectivity by inhibiting the formation of matured virus particles. Lopinavir has a short half-life time, therefore, to increase its half-life is used together with Ritonavir. Ritonavir, a cytochrome CYP3A4 inhibitor, inhibits the metabolism of Lopinavir by inhibiting cytochrome P450 and functions as a pharmacokinetic enhancer of Lopinavir [36]. Ritonavir/Lopinavir indicated antiviral effects against MERS-CoV and SARS-CoV-1 by inhibiting 3-chymotrypsin-like protease activity [37]. A randomized, open-label control study with patients with severe COVID-19 showed that no difference was seen in clinical improvement between the two groups treated with Ritonavir/Lopinavir and standard treatment. However, the secondary results of the study exhibited that in Ritonavir/Lopinavir group compared to those in the standard treatment group, patients had a shorter stay in the intensive care unit (ICU) and 28-day mortality was numerically lower in they [38].

#### Arbidol

Arbidol is a nonnucleoside antiviral agent that inhibits fusion between lipid envelope of the virus and the cell membrane of a host, thereby preventing virus entry into host cells. In addition, Arbidol can enhance the immune system of the host by inducing the production of INFs and activating the macrophages. It is found that Arbidol has an antiviral effect on a variety of viruses like a respiratory syncytial virus, hepatitis C and B viruses, influenza virus, and adenovirus [39]. A recent in vitro study showed that Arbidol has the ability for the inhibition of the infection of SARS-CoV-2 with EC_50_ of 4.11 μM [40]. In a retrospective trial by Deng and coworkers revealed that negative conversion rate of SARS-CoV-2 test in days 7 and 14 is significant elevated in patients treated with a combination of Ritonavir/Lopinavir plus oral Arbidol than patients treated with only Ritonavir/Lopinavir. In addition to, combination therapy remarkably improved the chest CT scans in 7-day [41].

#### Chloroquine/Hydroxychloroquine

Chloroquine and Hydroxychloroquine (HCQ) act as antimalarial and autoimmune disease drugs. Recently, their function as potential antiviral drugs has been reported. These two drugs are weak bases, which enter cells and accumulate in the endolysosomes and other acidic organelles, thereby elevating endosomal pH and preventing viral fusion into the cell. They also interfere with the terminal glycosylation of receptor ACE2 [42]. In addition, these two drugs can interfere with the proteolytic processing of M protein, resulting in control of the pro-inflammatory cytokines storm that occurs in late-phase COVID-19 patients [43]. The antiviral activity of Chloroquine (EC_50_ = 1.13 μM) on Vero E6 cells infected with SARS-CoV-2, was indicated in an in vitro study [16]. A recent clinical study of more than 100 patients with COVID-19 revealed that Chloroquine is superior to the control group in decreasing period symptoms, shortening disease period, and promoting coronavirus-negative conversion [44]. A recent in vitro trial indicated that Hydroxychloroquine with EC_50_ of 0.72 μM is more powerful compared to Chloroquine [43]. An open-label nonrandomized clinical study exhibited that Hydroxychloroquine in combination with Azithromycin can act as an alternative therapeutic approach to treat COVID-19 [45]. It is found that azithromycin enhanced the effectiveness of Hydroxychloroquine in reducing the viral load [46]. However, some clinical studies reported adverse effects, including prolonged QT interval, death, and transfer to intensive care in COVID-19 patients treated with Chloroquine/Hydroxychloroquine and azithromycin [47–50].

#### Recombinant soluble ACE2

ACE2, a transmembrane protein, is a carboxypeptidase and part of the renin-angiotensin system (RAS). ACE2 as a regulator of the RAS protects diverse tissues such as the lung by hydrolyzing angiotensin II to angiotensin 1–7 and angiotensin I to angiotensin 1–9. It is highly expressed in lungs, heart, kidneys, and small intestine and acts as the main receptor for SARS-CoV-2. Coronavirus binds to the ACE2 by its spike protein to enter into the host cells. The ACE2 downregulation by virus binding leads to loss of RAS homeostasis which then drives the severity of the disease [51]. A recent study suggested that an excess of soluble ACE2 would neutralize infection of SARS-CoV-2 via binding spike proteins and inhibiting virus-host membrane fusion [52]. Therefore, the utilization of recombinant human ACE2 to neutralize SARS-CoV-2 before it can bind to the membrane-bound ACE2 is explored as a therapeutic option. Recently, the therapeutic potential of human recombinant soluble ACE2 (hrACE2) to prevent primary SARS-CoV-2 infection, has been shown by Monteil et al. [53]. hrACE2 not only inhibits the early entry of SARS-CoV-2 into host cells but also protects COVID-19 patients from severe acute lung failure [54].

#### Azithromycin

Azithromycin is an antibiotic that exhibits antiviral and immunomodulatory properties, hence considered a potential treatment for COVID-19. Azithromycin acts in the treatment of COVID-19 by several potential mechanisms [55]. (1) Azithromycin binds to the ganglioside-binding site of SARS-CoV-2 spike protein and inhibits the binding of the virus to gangliosides on the host membrane. (2) Azithromycin can impair the endocytosis process and lysosomal protease action by increasing in lysosomal pH (3). Azithromycin suppresses the activation of CD4^+^ T-cells in lymphocytes. (4) Azithromycin can shift the polarization of alveolar macrophages to their anti-inflammatory phenotype and enhance apoptosis. (5) Azithromycin decreases the generation of pro-inflammatory chemokines and cytokines. (6) Azithromycin may suppress lung fibrosis through its antifibrotic activity in fibroblasts. As mentioned above Azithromycin has mostly been administered with Hydroxychloroquine to treat COVID-19 [45].

#### Ivermectin

Ivermectin is a broad-spectrum drug with anti-cancer, antiviral, and antimicrobial properties that has been utilized for the treatment of several infectious diseases. Recently, an in vitro study showed that Ivermectin acts as a powerful inhibitor of the SARS-CoV-2 in the Vero/hSLAM cells. In this study, a 5000-fold reduction in virus RNA levels than the control was observed. The speculated mechanism of the antiviral effect of Ivermectin against COVID-19 is the inhibition of importin α/β1 receptor. Importin, the main class of soluble transport receptors, is responsible for transporting viral proteins into the nucleus of the host cell [56]. In 2021, meta-analyses were done on 18 randomized controlled treatment trials. The findings showed that the use of Ivermectin to treat COVID-19 resulted in significant reductions in mortality, time to viral clearance, and time to clinical recovery. Moreover, data obtained from many controlled prophylaxis trials showed significantly decreased risks of contracting COVID-19 with the regular use of Ivermectin. These trials identified Ivermectin as an oral agent effective in all phases of COVID-19 [57]. However, a phase 3, double-blind, randomized, placebo-controlled trial indicated that none of the Ivermectin, Fluvoxamine, and Metformin drugs prevented hypoxemia occurrence, hospitalization, and death related to COVID-19 [58]. In addition, a meta-analysis done on 25 randomized controlled trials in 2022 indicated that Ivermectin doesn't decrease the risk of mortality risk and the risk of mechanical ventilation requirements [59].

#### Nitazoxanide

Nitazoxanide, an antiviral prodrug, is metabolized quickly to the active metabolite tizoxanide, this metabolite is safe and free of mutagenic effects. Previously, antiviral activity of Nitazoxanide against coronaviruses has been shown [60]. Wang et al. indicated that Nitazoxanide can suppress SARS-CoV-2 with a 2.12 μM concentration in Vero E6 cells [16]. A review on possible mechanisms of Nitazoxanide for repurposing in COVID-19 showed that it has the ability for blocking the entry of SARS-CoV-2 and inhibit its multiplication, prevent the cytokine storm, and amplify the host's innate antiviral response. Nitazoxanide may also protect the lung and prevent multiple organ failures [61].

#### Camostat mesylate

Camostat mesylate, a serine protease inhibitor, is a powerful inhibitor of TMPRSS2 and has been proposed as a potential antiviral agent against SARS-CoV-2. In vivo and in vitro trials have exhibited that Camostat mesylate blocks virus‐cell membrane fusion and therefore the replication of the virus [62].

#### Paxlovid

Paxlovid is an antiviral drug developed by Pfizer, which was approved by FDA on December 22, 2021, for treating patients with mild to moderate COVID-19. Paxlovid is an oral drug and 3C-like protease inhibitor; its use should be started during the first days of the disease and should be continued every 12 h for 5 days. Paxlovid is composed of the combination of PF-07321332 with ritonavir: PF-07321332 by inhibiting the protease of the SARS-CoV-2 prevents its replication. Ritonavir inhibits the rapid degradation of PF-07321332 in the body and causes PF-07321332 to remain in the body longer [63, 64]. Clinical research has indicated that if Paxlovid is initiated within the first three to five days of the symptoms onset in high-risk patients for the progression of the disease, it has the highest effect and prevents up to 89% of severe illness, hospitalization, and death [65]. The efficacy and safety of Paxlovid for COVID-19 are indicated in a meta-analysis conducted in 2022 [66]. Mutation of SARS-CoV-2 3CLpro led to resistance against Paxlovid [67].

### Biologic agents

Biologic agents, including monoclonal antibodies, convalescent plasma (SARS-CoV-2-specific neutralizing antibodies), hyperimmune sera, and exogenous surfactant delivery have attracted many notices during the outbreak of COVID -19 disease.

#### Monoclonal antibodies

Monoclonal antibodies (mAbs), because of their exceptional specificity to the virus, and their ability to coordinate the immune defense, are one of the most promising prophylactic and therapeutic tools to fight against viral infections. mAbs uses two pathways to neutralize COVID-19 infections [68]: (1) by targeting surface spike glycoprotein of SARS-CoV-2, (2) through targeting pro-inflammatory cytokines and chemokines involved in the SARS-CoV-2 infection (Fig. 2).

Interleukin-6 (IL-6), a pleiotropic cytokine with a pro-inflammatory activity, is a critical factor for the activation of the signal transduction pathway resulting in cytokine release syndrome (CRS). The cytokine storm and enhanced level of IL-6 in the blood are considered predictive of a fatal outcome in severe COVID-19 patients. IL-6 by binding to transmembrane receptors (mIL-6R) and soluble receptors (sIL-6R) activates the inflammatory response. Tocilizumab, an anti-IL-6R monoclonal antibody, binds to both receptors of IL-6 and effectively prevents the signal transduction pathway of IL-6. A retrospective study indicated that severe COVID-19 patients treated with Tocilizumab experienced clinical improvements, including fast defervescence, improved respiratory function, and discharged from the hospital without important adverse events [5, 69]. Leronlimab is a humanized mAb and a C–C chemokine receptor type 5 (CCR5) antagonist. The CCR5 is located on T regulatory cells, dendritic cells, and macrophages, to mediate chemotaxis in response to its ligands (CCL3, CCL4, and CCL5) [70]. In SARS-CoV infection, macrophages and airway epithelial cells express high levels of chemokine ligand 5 (CCL5). Leronlimab by competitively binding to the CCR5 inhibits CCL5, thereby reducing the downstream release of pro-inflammatory cytokines and inhibiting the migration of T-regulatory cells to the site of infection are occurred [71]. A small study showed that Leronlimab decreases the increased plasma levels of IL-6 and CCL5, and normalized CD4/CD8 ratios in severe COVID-19 patients [72].

CR3022 is a SARS-CoV receptor-binding domain (RBD)-specific mAb, which can bind to RBD of SARS-CoV-2 spike proteins. This binding can be established because of the lack of overlap between the ACE2 receptor-binding motif and the antibody’s epitope. In contrast to Yuan et al., Tian and colleagues found that CR3022 bound efficiently with SARS-CoV-2 RBD (KD = 6.3 nM), suggesting that CR3022 alone or in combination with other neutralizing antibodies might have the capability to prevent and treat of COVID-19. On the other hand, CR3022 is highly conserved among several coronaviruses and can be effective for COVID-19 treatment alone or in combination with other neutralizing antibodies [73, 74]. LY-CoV555 (Bamlanivimab) is a powerful anti-spike neutralizing mAb that specifically binds with various epitopes on the spike proteins of SARS-CoV-2. LY-CoV555 has been related to a reduction in viral load and frequency of emergency department visits and hospitalizations in COVID-19 outpatients [75]. LY-CoV1404 (bebtelovimab) is a fully human immunoglobulin G1(IgG1) mAb targeting the RBD of SARS-CoV-2 spike proteins. LY-CoV1404 neutralizes all SARS-CoV-2 variants [76]. Casirivimab plus Imdevimab are two human mAbs that bind to non-overlapping epitopes of RBD of SARS-CoV-2 spike protein [77]. Sotrovimab is a mAb that was identified from a SARS-CoV in 2003. This mAb binds to a conserved epitope in the spike protein RBD [78]. Sotrovimab and BRII-196 plus BRII-198 mAb cocktail potently prevent the replication of SARS-CoV-2 and has indicated effectiveness among COVID-19 outpatients for inhibiting disease progression to hospitalization or death [79].

In a meta-analysis of randomized controlled trials, it was shown that neutralizing mABs can help decrease the risk of hospitalization or emergency department visits in outpatients with COVID‐19 [80]. On August 31, 2021, was suggested that mAb therapy be administered based on a risk-based approach. For example, adolescents over 12 years of age with mild to moderate COVID-19 are at high risk of disease progression and hospitalization. mAbs can be used as post-exposure prophylaxis in those who are at high risk of exposure to COVID-19. After the emergence of the Omicron variant, Casivimab/Imdevimab and Bamlanivimab/Etesevimab became probably ineffectual as post-exposure prophylaxis or treatment [81].

Regrettably, SARS-CoV-2 has the potential to escape an efficient response, and mAbs capable to control mutating variants must be developed. Mutations that confer resistance to mAbs usually occur in the RBD and N-terminal domain of SARS-CoV-2 S-protein shown by using a recombinant chimeric VSV/SARS-CoV-2 reporter virus [82]. The E484K substitution in the RBD is one of the most frequent mutations to escape mAbs [83]. Recently, this mutation has been indicated to escape mAbs formerly approved for emergency use by the Food and Drug Administration [84, 85]. It seems that a useful solution to suppress the emergence of antibody resistance and increase the efficiency of mAbs in SARS-CoV-2 infection is to use a mixture of mAbs that target distinct epitopes on the RBD [82, 86, 87].

Sotrovimab is a mAb that seems to be fully effective in the treatment of the Omicron variant. Sotrovimab doesn't block the ACE2 but targets “non-receptor-binding motif (RBM)” epitopes that are common among many sarbecoviruses such as SARS-CoV [88, 89]. Therefore, this mAb avoids the likely antigenic “shift” imposed via the Omicron variant [90]. Recently, an ACE2-Fc fusion protein, SI–F019, presented as a novel therapeutic agent against dominant variants including Omicron that not only isn't its neutralization effectiveness not lost but also is indeed strengthened by the mutations of dominant variants [91].

#### Convalescent plasma

Convalescent plasma, i.e. immune plasma collected from individuals recovered from the infectious disease, provides immediate passive short-term immunity to susceptible individuals. Convalescent plasma is a type of therapy or ideal post-exposure prophylaxis for COVID-19. The suppression of viremia by immunoglobulins is an explanation for the effectiveness of convalescent plasma therapy (Fig. 2). The use of convalescent plasma as a neutralizing and/or immunomodulatory agent has been previously reported in Influenza, Ebola, MERS, and SARS patients [92]. The success of utilizing convalescent plasma therapy for COVID-19 patients has been reported in several studies. Shen et al. used convalescent plasma containing SARS-CoV-2-specific antibodies to treat 5 seriously ill COVID-19 patients with ARDS. Following receipt of convalescent plasma, the clinical symptoms remarkably improved [93]. In another study, convalescent plasma was transfused into ten severe COVID-19 patients to rescue them. After the transfusion of convalescent plasma, a fast increase in the level of neutralizing antibodies was found. All ten patients presented improved clinical symptoms, including enhanced lymphocyte counts and reduced C-reactive protein. In addition, in 7 patients who had viremia before convalescent plasma transfusion, viral load was not detected [94]. While data are restricted, they suggest clinical advantages in terms of a decrease in viral loads, resolution of the radiological changes, improved survival, and reduced mortality.

Nowadays, the ineffectiveness of convalescent plasma for hospitalized patients with moderate to severe COVID-19 has been proven by several randomized trials [95, 96]. However, convalescent plasma can be useful for early disease when transfused in high-titer to outpatients within 72 h after the onset of symptoms [97, 98].

#### Hyperimmune sera

The therapy with hyperimmune sera is a type of passive immunotherapy that has been used recently as adjunctive therapy to antivirals in European countries affected by COVID-19 [99]. The aim of passive immunization treatment is the enhancement of immune response and the prevention of disease progression [100]. Hyperimmune sera have polyclonal antibodies, predominantly of heterologous immunoglobulin G (IgG), which can be applied to treat viral infections [101]. The use of hyperimmune serum is strongly feasible when vaccines aren't available or aren't entirely accepted by people [102]. The hyperimmune sera have some advantages over COVID-19 convalescent plasma including, easier preservation, an easier administration route, and a smaller reinfusion volume. Moreover, the low cost of hyperimmune serum production and the more diversification of neutralizing antibodies in it makes it preferable to mAbs [103].

#### Exogenous surfactant delivery

Due to the binding S protein of SARS-CoV-2 to ACE2 receptor of alveolar type 2 (AT2) lung cells and the lysis of these cells by the virus, it seems reasonable to reduce the synthesis and secrete endogenous surfactant in COVID-19 patients. Therefore, exogenous surfactant delivery can mitigate its activity deficits. The obtained data from the study of Pive et al. indicated that liquid surfactant delivery to patients with severe COVID-19 ARDS is feasible and well-tolerated [104].

### Anti-inflammatory agents

Hyper-inflammation and cytokine storm are recognized as crucial players in the progression of COVID-19 disease toward severe interstitial pneumonia, ARDS, and coagulopathies. Therefore, finding treatments that target both the virus and consequent hyper-inflammation is essential for the effective treatment of the CCVID-19 disease. Corticosteroids, intravenous immunoglobulin (IVIG), Janus kinase (JAK) inhibitors, and Colchicine, are anti-inflammatory treatments used to treat COVID-19.

#### Corticosteroids

Systemic corticosteroids are immunosuppressive agents that are used broadly to treat patients with severe viral ARDS. Corticosteroids by using immunosuppressive and anti-inflammatory properties, minimize the injury created by viruses in the body. The anti-inflammatory property of corticosteroids is related to the suppression of pro-inflammatory genes via signal transduction through their steroid receptors. During outbreaks of MERS-CoV and SARS-CoV, systemic corticosteroids have been used. Although, clinical evidence has revealed that corticosteroid therapy delayed the viral clearance in these infections. Similarly, corticosteroids in patients with influenza pneumonia increased secondary infection rates and mortality [105]. The utilization of corticosteroids for COVID-19 infection management is widely discussed. A recent guideline from the WHO doesn't recommend the utilize of corticosteroids if COVID-19 is suspected because they can prevent the generation of important antiviral mediators (especially, type I and III INFs) [106]. Wu C et al. reported that corticosteroid decreases fatality when utilized in COVID-19 patients with ARDS [107], although several retrospective trials reported enhanced fatality in treated patients with the corticosteroid [108]. Recently, a recovery trial indicated that Dexamethasone decreased mortality in patients who required oxygen treatment with and without invasive mechanical ventilation [109].

Budesonide is an inhaled corticosteroid with broad anti-inflammatory properties that recommend for chronic respiratory diseases. The inhibitory effect the certain inhaled corticosteroids on viral replication of SARS-CoV-2 have been shown [110]. Also, the expression of the receptors utilized to enter cells is downregulated by these corticosteroids [111]. The efficiency of inhaled budesonide is investigated in outpatients with COVID-19 mild symptoms in two open-label trials. Results showed that initiation of inhaled Budesonide in these patients may decrease the requirement for necessary care or hospitalization and decrease the time for recovery [112, 113].

Immunomodulatory therapy, such as corticosteroids, may result in more frequent or severe secondary bacterial or fungal superinfections in hospitalized patients. In COVID-19 patients with ARDS on invasive mechanical ventilation, a remarkably greater rate of superinfections has been reported [114, 115]. Søvik et al. indicated that whereas Dexamethasone increases survival in severely ill COVID-19 patients, it also appears to enhance the risk of clinically relevant superinfections [116].

#### Fluvoxamine

Fluvoxamine is an approved serotonin reuptake inhibitor (SSRI) for the treatment of depression and obsessive–compulsive disorder. The anti-inflammatory effect of Fluvoxamine was found in a murine sepsis model. It binds to the sigma-1 receptor in immune cells, leading to decreased generation of inflammatory cytokines [117]. In addition, Fluvoxamine decreased the expression of the inflammatory gene in an in vitro study of human macrophages and endothelial cells [118]. In 2020, Lenze et al. indicated that Fluvoxamine could prevent clinical deterioration in early-stage COVID-19 outpatients [119]. Furthermore, Hoertel et al. suggested that the utilization of antidepressants, such as SSRIs and serotonin-norepinephrine reuptake inhibitors (SNRIs), may be related to decreased risk of intubation or death in hospitalized patients with COVID-19 [120]. Because the human lung has a high level of serotonin transporter expression [121], Fluvoxamine may affect the lung function of patients with COVID-19 [122]. In 2021, Seftel and his colleague performed a prospective cohort study of Fluvoxamine in outpatients infected with SARS-CoV-2. They indicated that the incidence of hospitalization was sex in the observation-alone group and zero in the Fluvoxamine-treated group [123]. Therefore, on April 23, 2021, Fluvoxamine was added to the US NIH COVID-19 Guidelines Panel. A meta-analysis done in 2022 indicated that three oral drugs (Fluvoxamine, Molnupiravir, and Paxlovid) were efficient in decreasing hospitalization rates and mortality in COVID-19 patients. In addition, these drugs exhibit well safety because did not enhance adverse events occurrence. Therefore, they possess the potential to be a promising and breakthrough treatment for COVID-19 [124].

#### Anakinra

Anakinra, a recombinant interleukin (IL)‐1 receptor antagonist, prevents IL-1α and IL-1β from attaching to IL-1 type I receptors, thereby neutralizing their activity in immune or/and auto-inflammatory processes. Anakinra has a short half-life which enables it to quickly discontinue its action regarding secondary infections or adverse reactions, therefore, making it suitable for critically ill patients [125]. In addition, the inhibition of IL-1 is related to a decrease in the dysfunction of endothelial and the alteration of microvascular, which seems key in COVID-19–related thromboembolic events [126]. Anakinra is an approved treatment for cytokine storm syndromes and hyper-inflammatory conditions, including cytokine release syndrome, macrophage activation syndrome, and Still's disease [127]. Recently, the effectuality of Anakinra in severe COVID-19 patients has been reported in several small studies. In a cohort study, a significant reduction was observed in the requirement for invasive ventilation and the fatality rate in severe COVID-19 patients, who received Anakinra [125].

#### Granulocyte-macrophage colony-stimulating factor (GM-CSF) inhibitors

GM-CSF is a pro-inflammatory cytokine and myelopoietic growth factor that has a key role in a wide range of immune-mediated diseases. The function of GM-CSF as a pro-inflammatory signal promotes macrophages to launch an immune cascade, which eventually leads to the damage of tissue [128]. It is believed that GM-CSF is a critical operator of lung inflammation in severe COVID-19 pneumonia that operates upstream of other chemokines and pro-inflammatory cytokines [129]. Anti-GM-CSF mAbs can decrease inflammation from COVID-19 by repressing this signaling axis upstream and consequently reducing the downstream generation of various pro-inflammatory mediators involved in the pathogenesis of COVID-19 [130]. Otilimab, Namilumab, Lenzilumab, and Gimsilumab by targeting GM-CSF directly and preventing its interaction with cell surface receptors, neutralizing the biological function of GM-CSF [131, 132]. Mavrilimumab by targeting the alpha subunit of the receptor of GM-CSF blocks its intracellular signaling [133].

#### Intravenous immunoglobulin (IVIG)

IVIG is a blood-derived product that is prepared from the plasma of healthy donors and is usually utilized as supportive treatment. IVIG contains a pool of polyclonal immunoglobulin G, which is frequently used as an immunotherapeutic molecule to treat different autoimmune and inflammatory diseases. Favorable results of previous works on MERS and SARS suggested the use of IVIG for managing patients with severe COVID-19. The efficacy of IVIG can be enhanced by utilizing IgG antibodies that are collected from recovered COVID-19 patients in the same city or the surrounding region, to enhance the chance of neutralizing SARS-CoV-2 [134]. High-dose IVIG therapy plays a role in modulating immune inflammation and is considered to increase passive immunity. It is still unclear how IVIG helps patients with severe COVID‐19. The decrease in inflammatory mediators after IVIG therapy, have reported in several studies, therefore they suggested that IVIG could target cytokine storm in patients with severe COVID‐19 by complement scavenging, inhibition of effector T helper cells (Th1/17) cells, expansion of regulatory T-cells, and suppression of the activation of innate immune cells [135]. Shi et al. reported that timely initiation of plasma exchange along with IVIG in severe COVID-19 patients can treat them without mechanical ventilation or intensive supportive treatment [136]. Similarly, in other studies, the early utilize of high-dose IVIG in COVID-19 patients was found to be effective in improving the clinical condition, preventing the progression of pulmonary lesions, and reducing the use of mechanical ventilation and the hospitalization period [137]. Therefore, the timing of administration of IVIG is the main factor determining the outcome of IVIG therapy, it should be administered before the initiation of systemic injury.

#### Janus kinase (JAK) inhibitors

JAK inhibitors are potent inhibitors of the JAK family enzymes that interfere with the JAK-STAT signaling pathway [138]. The JAK/STAT pathway mediates the effect of various molecules such as interleukins, IFNs, and growth factors [139]. JAK inhibitors have dual anti-inflammatory and anti-viral effects in COVID-19. They inhibit the signaling of many pro-inflammatory cytokines involved in the cytokine storm such as IL-6, and also block entry of SARS-CoV-2 to alveolar type 2 alveolar epithelial cells (Fig. 2) [140]. SARS-CoV-2 can enter into the cell through endocytosis and invade the cells. The AP2-associated protein kinase 1 (AAK1), a member of the numb-associated kinase (NAK) family, is a known regulator of clathrin-mediated endocytosis. Baricitinib is a JAK1/2 inhibitor, which utilized in rheumatoid arthritis to prevent the production of pro-inflammatory cytokine. Baricitinib is also a NAK inhibitor with a particularly high affinity for AAK1 that prevents the passage of the virus into cells. Recently, Baricitinib is suggested as a useful cure for pneumonia during COVID-19 by Richardson et al. They reported that Baricitinib on therapeutic dosing can inhibit AAK1 and cyclin G-associated kinase (another regulator of endocytosis of virus) functions [141]. The other inhibitor JAK is Tofacitinib, which inhibits JAK1 and JAK3 selectivity and has functional selectivity for JAK2. The inhibitory action of Tofacitinib on inflammatory cascade pathways may improve advanced, inflammation-driven lung injury in hospitalized COVID-19 patients. Guimarães et al. reported that Tofacitinib caused a lower risk of respiratory failure or death through day 28 than placebo in patients hospitalized with COVID-19 [142].

#### Colchicine

Colchicine, an anti-inflammatory and immunomodulatory agent, is approved for gout and familial Mediterranean fever. Recently, Colchicine has obtained attention in the management of some complications of COVID-19 infection. The main mechanism of Colchicine anti-inflammatory action is related to its ability to inhibit activation of Nod‐like receptor protein 3 (NLRP3) inflammasome. NLRP3 inflammasome is a main pathophysiological component in the ARDS development in patients with COVID-19. The inhibition of the NLRP3 inflammasome by Colchicine results in suppressing the activation of caspase-1 and the subsequent release of IL-1β and IL-18 [143]. Several trials have been registered for COVID-19 treatment via conventional therapeutic doses of colchicine (NCT04326790, NCT04328480, NCT04322682, NCT04322565).

### Herbal agents

Traditional Chinese medicine (TCM) alone or in combination with Western medicine was considered as an alternative treatment strategy to treat COVID-19, based on historical experience and anecdotal evidence of the prevention of H1N1 influenza and SARS. The utilization of TCM in COVID-19 patients in China presents promising outcomes in the improvement of clinical symptoms and the reduction of deterioration, recurrence, and mortality rates. The mechanism of Chinese herbal medicine (CHM) on COVID-19 is multi-component, multi-pathway, and multi-target. The primary mechanisms are direct anti-viral activity, anti-inflammatory action, the regulation of the immune system, and the protection of target organs. Atractylodis Macrocephalae Rhizoma (Baizhu), Fructus forsythia (Lianqiao), Lonicerae Japonicae Flos, Saposhnikoviae Radix (Fangfeng), Glycyrrhizae Radix Et Rhizoma (Gancao), and Astragali Radix (Huangqi) are some CHM that were broadly used during COVID-19 outbreak in China [144]. However, precise clinical trials on large populations of patients COVID-19 are required to confirm the preventive effect of CHM.

Other treatments against SARS-CoV-2 are listed in Table 1. In addition, to further assess the efficacy of drugs on COVID-19, several pre-clinical and clinical studies of different potential treatments have been performed and their results are listed in Table 2. The SARS-CoV-2 clearance, time to clinical improvement, length of stay in the Ward or ICU, need for mechanical ventilation, and mortality of COVID-19 patients have been assessed in these studies. Moreover, the probability of secondary infection, the need for ICU admission, symptoms resolution, and results related to CT-scan in these patients have been studied.

**Table 1:** List of treatments for COVID-19

Therapeutic agent	Type of therapeutic agent	Common uses	Mechanism of action	References
Melatonin	Hormone with anti-viral, anti-oxidant, and anti-inflammatory properties	Broad-range of diseases such as rheumatoid arthritis, diabetes, sleep disturbances, chronic fatigue syndrome, infectious diseases, etc	The reduction of infection-associated oxidative stress, the inhibition of NLRP3 inflammasome, the promotion of adaptive immune activity, and the decrease of immunosuppression induced by sleep deprivation and chronic stress	[169]
Sarilumab	Human mAb against IL-6 receptor	Rheumatoid arthritis	By inhibiting IL-6 receptor signaling	[170]
Siltuximab	Anti-IL-6 mAb	Multicentric Castleman disease	The prevention of the binding of IL-6 to both soluble and membrane-bound IL-6 receptors and the inhibition of IL-6 signaling	[170]
Dapagliflozin	Sodium-glucose cotransporter-2 inhibitor	Diabetes and heart failure	The increase of the ACE2 level, the reduction of the viral load, and the prevention of the lowering of cytosolic pH	[171]
Fedratinib	JAK2 inhibitor	Myelofibrosis	The prevention of the deteriorating results of Th17 associated cytokine storm by inhibiting the generation of some Th17 signature cytokines	[172]
Ruxolitinib	JAK1/2 inhibitor	Polycythemia vera and myelofibrosis	The reduction of systemic inflammation by mitigating exuberant cytokine storm	[173]
Acalabrutinib	Bruton’s tyrosine kinase (BTK) inhibitor	B-cell malignancies	Modulation of inflammation-promoting signaling	[174]
Ibrutinib	BTK inhibitor	B-cell malignancies	The reduction of inflammation	[175]
Zanubrutinib	BTK inhibitor	Mantle cell lymphoma	Modulation of inflammation-promoting signaling	[176]
Statins	3‐hydroxy‐3‐methyl‐glutaryl‐CoA reductase inhibitors	Atherosclerotic cardiovascular disease	The modulation of virus entry by acting on the CD147 and ACE2 receptors, the regulation of virus replication or degradation by inducing autophagy activation, and the limitation of cytokine storm by inhibiting NLRP3 inflammasomes and NF‐κB	[177]
Cyclosporine	Calcineurin inhibitor	Rheumatoid arthritis, systemic lupus erythematosus, or interstitial lung disease	The prevention of the replication of SARS-CoV-2, un-controlled inflammatory response, and acute lung injury	[178]
Sofosbuvir + Daclatasvir, Sofosbuvir + Ledipasvir	RdRp inhibitors	Hepatitis C	The termination of the RNA synthesis process by incorporating into the RNA chain	[179]
Galidesivir	RdRp inhibitor	Ebola	The inhibition of viral replication by premature termination of RNA transcription	[180]
Leflunomide	Human dihydroorotate dehydrogenase (DHODH) inhibitor	Autoimmune diseases	Direct targeting the DHODH (host de-novo pyrimidine synthesis enzyme), and hence the inhibition of viral RNA genome replication	[181]
Adalimumab	mAb, tumor necrosis factor-α inhibitor	Hidradenitis suppurativa	The inhibition of the TNF-α cytokine	[182]
Bevacizumab	mAb, vascular endothelial growth factor (VEGF) inhibitor	Cancer	The improvement of oxygen perfusion and anti-inflammatory response by inhibiting the VEGF and its receptor	[183]
Camrelizumab	mAb, programmed cell death protein 1 (PD‐1) immune checkpoint inhibitor	Relapsed or refractory classical Hodgkin lymphoma	The prevention of T cell death, the regulation of cytokine production and the reduction of organ dysfunction by blockade of PD‐1	[184]
Eculizumab	mAb, anti- complement 5 (C5)	Paroxysmal nocturnal hemoglobinuria, and atypical (complement-mediated) HUS (aHUS)	The inhibition of complement-mediated acute kidney injury by suppressing C5	[185]
Sirolimus, also known as Rapamycin	The mammalian target of rapamycin (mTOR) signaling pathway inhibitor	Tuberous sclerosis	Inhibits the proliferation of effector T-cell and promotes the accumulation of T_reg_. It can decrease damage from the immune during COVID-19 infection	[186]
Telmisartan, Losartan	Angiotensin receptor blockers	Hypertension	The binding to the AT1 receptor and its irreversible block, the prevention of SARS‐CoV‐2 interaction with the ACE2 catalytic site	[187]
Valsartan	Nonpeptide angiotensin receptor antagonist	Chronic symptomatic heart failure	The block of the receptor of angiotensin II and maximize the anti-inflammatory properties of an increased natriuretic peptide system	[188]
Captopril	ACE inhibitor	Hypertension	The increase of ACE2 levels and promote the anti-inflammatory RAS axis in the lung	[189]
N-Acetylcysteine (NAC)	Glutathione precursor with anti-inflammatory and antioxidant properties	Substance use disorders	The lowering mucus viscosity and the regulation of redox state due to restoring thiol pools	[190]
Bromhexine	TMPRSS2 inhibitor	Mucolytic cough suppressant	The inhibition of TMPRSS2-specific viral entry	[191]
Vitamin C	Antioxidant	Viral infections	The inhibition of cytokine storm, the promotion of innate antiviral immunological response, the prevention of excessive inflammatory response, the reduction of lung inflammation and injury, and the restoring of endothelial function	[192]
Vitamin D	Secosteroid	Supplements	The inhibition of expression of inflammatory cytokines like L-1α, IL-1β, TNF-α	[193]
Curcumin	Natural polyphenolic compound	Food consumption	The inhibition of the entry of the virus, the suppression of assembling of the virus and viral protease, and the modulation of different cellular signaling pathways	[194]

**Table 2:** Pre-clinical and clinical studies of different potential treatments in COVID-19 patients

	SARS-CoV-2 Clearance			Time to Clinical improvement			Length of stay		Mechanical Ventilation		Mortality			Secondary infection	ICU admission rate	Symptoms Resolution	Critical progressing	CT-Scan	*Lab data	Other	References
	Rate until day	Time to clearance	Viral load	7—ordinal scale	based on study	Oxygen support	Ward	ICU	Duration in days	Requirement rate	28-day mortality	Simple death rate	Time to death								
Novaferon	✓	✓																			[195]
INF β-1a				✓			✓	✓	✓		✓										[196]
Baloxavir Marboxil and Favipiravir		✓		✓						✓											[197]
Hydrocortisone				✓						✓				✓							[198]
Corticosteroid												✓			✓						[199]
Favipiravir		✓			✓																[200]
TCM														✓		✓					[201]
Granulocyte colony- stimulating factor			✓	✓		✓	✓				✓						✓				[202]
Kaletra				✓			✓		✓		✓										[38]
Plasma							✓					✓					✓				[203]
Azithromycin							✓	✓				✓			✓						[204]
HCQ and Azithromycin	✓						✓					✓									[45]
HCQ							✓				✓						✓				[205]
HCQ									✓				✓								[206]
Azithromycin					✓		✓				✓			✓							[207]
Azvudine	✓																	✓			[208]
Favipiravir	✓	✓										✓				✓	✓				[209]
Sovodac							✓	✓				✓									[210]
TCM																					[211]
Sovodac					✓		✓			✓		✓									[212]
INF β-1b					✓		✓			✓	✓				✓						[213]
Actemra												✓			✓						[214]
Arbidol		✓														✓					[215]
Combinational Regimen												✓					✓	✓			[216]
HCQ and Atazanavir							✓	✓		✓	✓				✓						[217]
Ruxolitinib		✓			✓				✓		✓	✓						✓	Cytokines/Lymphocyte recovery time		[173]
TCM																✓			CRP/ESR/Lymphocyte/WBC		[218]
Dexamethasone				✓					✓		✓										[219]
Methyl Prednisolone					✓	✓	✓					✓	✓					✓	CBC/CRP/ESR/IL-6/Ferritin/Troponin/D-Dimer/LDH/CPK		[220]
IFN β-1a				✓			✓	✓			✓										[221]
Bromhexine							✓			✓	✓				✓	✓			CRP/LDH/NLR		[222]
Colchicine				✓						✓		✓							Troponin/CRP		[223]
Leflunomide		✓					✓												CRP		[224]
Azithromycin							✓	✓				✓			✓		✓				[204]
HCQ					✓				✓		✓										[225]
EECOVERY study (Dexamethasone)									✓	✓	✓										[226]
Leflunomide		✓					✓										✓		CRP/Cytokines		[227]
METCOVID study (Methyl Prednisolone)							✓			✓	✓							✓			[228]
Remdesivir				✓		✓	✓			✓	✓			✓							[229]
ReciGen®			✓				✓	✓				✓				✓		✓			[230]
HCQ and Azithromycin				✓			✓	✓	✓											Remdesivir requirement rate!	[231]
IVIG												✓									[232]
Arbidol							✓			✓	✓				✓	✓		✓	WBC/ESR/SPO-2/Time to fever-free		[233]
HCQ		✓		✓		✓	✓					✓			✓				CRP/LDH/Ferritin/IL-6/D-Dimer	Vasopressor utilization /	[205]
Baricitinib and Remdesivir				✓		✓	✓		✓		✓								Clinical Status at day 15	Time to NEWS2 Score (2 or less)	[234]
Tocilizumab							✓			✓	✓				✓		✓				[235]
HCQ	✓	✓					✓					✓									[236]
Tocilizumab					✓	✓		✓	✓		✓			✓					CRP/Relation between Actemra dose and fever resolution	Vasopressor Utilization /	[237]
TCM					✓		✓												ALT/AST/Cr/CBC/Pt/IL-6/CRP		[238]
Ivermectin	✓															✓	✓			Anosmia	[239]
Fluvoxamine				✓		✓	✓		✓								✓				[119]
Remdesivir				✓	✓	✓	✓					✓								Duration of different modes of respiratory support	[240]
Anakinra							✓			✓	✓	✓							CRP	Clinical status assessed via WHO-CPS scoring	[241]
Enisamium				✓																	[242]
Vitamin C						✓	✓	✓	✓		✓						✓		IL-6/CBC/Procalcitonin/CRP	SOFA score/Vasopressor utilization	[243]
Curcumin						✓	✓							✓		✓	✓		Lab Data/Lymphocyte count/CRP	O_2_ Saturation	[244]
												✓							IL-1β, IL-6, TNF-α, IL-18		[245]
Zinc					✓					✓	✓										[246]
Calcifediol												✓			✓						[247]
Plasma	✓			✓		✓				✓	✓					✓	✓			SOFA score/Vasopressor utilization	[248]
							✓			✓		✓									[249]
BISCUIT Trial		✓					✓											✓	D-Dimer/CRP/Lymphocyte/NLR/Lymphocyte to CRP ratio	SHOKS-COVID Score system/Fever time/SPO-2/Respiratory rate/NEWS2 score	[250]
TCM					✓		✓													Clinical improvement rate	[238]
Sovodac																✓				Contrary to others, this trial was conducted on outpatients and Hospital admission rate was the outcome!	[179]
Pentoxyfylline							✓			✓		✓							LDH/Lymphocyte		[251]
HCQ and Azithro	✓				✓											✓					[252]
Favipiravir and Actemra																✓		✓	IL-6/Lymphocytes		[253]
HCQ				✓		✓	✓	✓		✓	✓									Vasopressor utilization	[254]
TCM																✓	✓	✓		ARDS development	[255]
Tocilizumab				✓		✓	✓		✓		✓			✓						SOFA score/Occurrence of thromboembolic events	[256]
Remdesivir				✓						✓		✓									[257]
Kaletra & Ribavirin & INF beta		✓	✓				✓				✓					✓			IL-6/TNF-α	NEWS2/SOFA	[34]
IFN-k		✓																✓	CBC/CRP/TNF-α/IL-22/IL-10/IL-8/IL-6/IL-1β /		[258]
IVIG & Methylprednisolone							✓	✓		✓										PaO_2_/FiO_2_ improvement	[259]
Actemra and Corticosteroid				✓		✓	✓		✓	✓		✓									[260]
SOLIDARITY trial							✓			✓		✓									[261]
Colchicine						✓	✓	✓				✓			✓				CRP/LDH/NLR ratio		[262]
Itolizumab																			IL-6/IL-1/TNF-α/Neutrophil/Lymphocyte		[263]

*Lab data: Laboratory reports measured in these studies

## Preventive agents

### Vaccines

The rapid development of an effective vaccine is an immediate need to protect the global community from the threat of mortality from COVID-19 disease. Since COVID-19 outbreak began, researchers around the world are working to develop a vaccine, so far there are 273 vaccine candidates in preclinical and clinical trials. COVID-19 vaccination, as part of the exit strategy, can provide a return to previous patterns of socializing, schooling, and working. At first, it was recommended that health workers, people in shielding groups, and people over 65 be vaccinated.

Since vaccine-induced immune response can result in disease, vaccine development for SARS-CoV-2 is accompanied by concern. By necessity, vaccine development for emerging infections will need a shorter flow from discovery to deployment, and thus predicting safety in early the process is important. The vaccine-induced immune response can either appear as an acute response to the vaccine itself or as the enhancement of disease after the infection of the virus [145]. It was found that vaccines targeting the RBD, S1, or S2 subunits of SARS-CoV-2 have high protective effects on COVID-19. Therefore, COVID-19 vaccines were designed and developed to weaken or disrupt the interactions of RBD or destabilize the S protein [54].

The various platforms are being adopted for COVID-19 vaccine development, including DNA, RNA, protein subunits, live attenuated viruses, inactivated viruses, virus-like particles, and non-replicating viral vectors [146]. As SARS-CoV-2 is new and there is a poor understanding of the nature of protective immune responses, it is uncertain that the vaccine development strategy will be successful. Thus, it is crucial to use different strategies and platforms to develop vaccines in parallel. RNA and DNA-based vaccines have several benefits in a pandemic situation. Their first benefit is the rapid development in the laboratory, due to no need for bio reactor culture techniques. The next benefit is the generation of a robust immune response by these two platforms [147]. Before the COVID-19 pandemic, because of the low stability and dubiety surrounding mRNA vaccine formulation, no mRNA vaccine candidate was successfully commercialized [148]. The mRNA delivery into the cytoplasm is essential; thus, different approaches have been utilized such as polyplexes, cationic nano-emulsions (CNEs), and lipid nanoparticles (LNPs) [149]. DNA vaccines are eukaryotic expression plasmid DNA that encodes target antigen protein. The transcription and translation of the antigen after the vaccine is taken up by host cells can produce immune responses in the body and thereby protect the host [150]. Vaccines based on viral vectors are live attenuated vaccines that use modified safe viruses like adenovirus as the vector to express the desired antigen(s). They have long-term stability that elicits effective and potent immune responses. Furthermore, they can be made on a large scale [151]. Live vector vaccine is a combination of the strong immunogenicity of live attenuated vaccine and the safety of subunit vaccine [152]. If the vector vaccine has already been exposed to the target virus, its effectiveness will decrease because of the previously presenting immunity against the vector [153].

Live attenuated-virus vaccines can be prepared by utilizing a virus with reduced pathogenesis. Although these vaccines stimulate the innate immune system, there is a probability that attenuated vaccine strain is recombined with wild viruses to create a pathogenic strain [154]. Live attenuated-virus vaccine mostly induces mucosal immunity to decrease the virus mucosal infection. Vaccines based on chemically or physically inactivated viruses have few safety concerns and express a broad range of native virus antigens. These vaccines are weak inducers of cytotoxic CD8^+^ T cells, a suitable property for an efficient COVID-19 vaccine [155]. The ability to trigger the toll-like receptors including TLR 3, TLR 7/8, and TLR 9 is an important advantage of attenuated and inactivated vaccines. Other advantages of these vaccines that make them suitable for vaccination include the induction of excellent B cell response, the preservation of the viral structure, rapid development, and site-directed mutagenesis [152]. Although inactivated vaccines are more stable than attenuated vaccines, the immune memory produced by inactivated vaccines is short-lived, which requires the inoculation of higher doses and the association of the inactivated virus with an adjuvant [156].

Most protein subunit vaccines have full-length spike protein of SARS-CoV-2 or portions of it that are used to induce neutralizing antibodies [157]. These antibodies inhibit viral genome uncoating and receptor binding. Subunit vaccines mostly induce CD4^+^ Th cells and are weak activators of CD8^+^ T-cells responses. The protein subunit vaccines needs to utilize along with adjuvants to enhance their immunogenicity. The safety of subunit vaccines is increased due to the absence of an entire virus, however, the return of toxicity is an important problem in these vaccines [158].

The array of spike proteins on the surface of virus-like particles crosslinks the receptor of B cells and activates B cells directly, unlike protein subunit vaccines. Also, virus-like particles need an adjuvant and repeated administration, like inactivated viral and protein subunit vaccines [159]. Virus-like particle is not capable of replicating or inducing infection due to the absence of genetic materials and does not need the protection of biosafety and special laboratory settings. Therefore, they are proper and safe models for vaccine design and viral molecular studies [160].

To date, from 108 candidate vaccines entered into human clinical phases, 8 candidates have been entered into clinical 4 phase, 19 vaccines into 3 phase, and 6 vaccines into phase 2/3.

Pfizer/BioNTech, named BNT162, is an mRNA-based vaccine, which encodes the RBD of the SARS-CoV-2 spike glycoprotein. The RBD antigen expressed by BNT162 is fused to a T4 fibritin-derived ‘foldon’ trimerization domain to enhance the immune response. Pfizer/BioNTech has announced efficacy of 95% [161]. Moderna vaccines, named mRNA-1273 and mRNA-1273.351, are other mRNA-based vaccines that encode for SARS-CoV-2 spike protein. The efficacy of Moderna has been announced 94.5% in the prevention of COVID-19 [162]. Sinovac (CoronaVac) is a vaccine based overall inactivated virus with an aluminum adjuvant. Sinovac vaccine is currently under phase 4 trials. Sinopharm is developing two inactivated vaccines that are under phase 3 and 4 trials. Preliminary results indicated that Sinovac’s SARS-CoV-2 vaccine generates antibodies that neutralize 10 strains of SARS-CoV-2 [163]. Sinopharm is collaborating with Wuhan Institute of Biological Products and Beijing Institute of Biological Products to develop these two vaccines. Sinopharm has now announced an efficacy of 79% [164]. AstraZeneca in collaboration with Oxford University develops a vaccine with an efficacy of 70% to prevent COVID19. AstraZeneca vaccine (ChAdOx1-S (AZD1222)) is currently under phase 4 trials and uses a non-replicating chimpanzee adenovirus to deliver spike protein of SARS-CoV-2 to elicit immune responses. The expressed spike protein on the surface of the virus particle, triggers both T-cell and antibody responses, which may be protective against COVID-19 [165]. Gamaleya, Sputnik V, is a vaccine based on two adenovirus vectors. Sputnik V is currently under phase 3 trials and reportedly shows 92% protection against COVID-19 [166]. To make both CanSino’s (Ad5-nCoV) and Janssen (Ad26.COV2) vaccines (both are currently under phase 4 trials), adenovirus vector-based vaccine platforms are used. Adenoviruses utilized to make these vaccines are inactivated because of the E1 gene deletion and its replacement with the spike gene [167]. Novavax vaccine (NVX-CoV237) consists of prefusion SARS-CoV-2 spike protein, harvested from genetically modified viruses. NVX-CoV237 efficacy is 86% against UK variant and 60% against South African variant [168] and is currently under phase 3 trials. Recombinant SARS-CoV-2 vaccines (Anhui Zhifei Longcom Biopharmaceutical/Institute of Microbiology, Chinese Academy of Sciences and West China Hospital, Sichuan University), mRNA vaccines of Curevac and ARCoV (Academy of Military Science (AMS), Walvax Biotechnology and Suzhou Abogen Biosciences), inactivated SARS-CoV-2 vaccine (Institute of Medical Biology/Chinese Academy of Medical Sciences), inactivated vaccine QAZCOVID-IN®-COVID-19 (Research Institute for Biological Safety Problems, Rep of Kazakhstan), inactivated SARS-CoV-2 vaccine (Shenzhen Kangtai Biological Products Co., Ltd.), inactivated vaccine of VLA2001 (Valneva, National Institute for Health Research, United Kingdom), nCov DNA vaccine (Zydus Cadila), SARS-CoV-2 vaccine formulation 1 with adjuvant 1, VAT00002 (Sanofi Pasteur /GSK), FINLAY-FR anti-SARS-CoV-2 vaccine (Instituto Finlay de Vacunas), EpiVacCorona vaccine based on peptide antigens (Federal Budgetary Research Institution State Research Center of Virology and Biotechnology "Vector"), whole-virion inactivated SARS-CoV-2 vaccine, BBV152 (Bharat Biotech International Limited), protein subunit vaccine CIGB-66, RBD with aluminum hydroxide as adjuvant (Center for Genetic Engineering and Biotechnology (CIGB)), recombinant protein vaccine of Nanocovax, aluminum as an adjuvant (Nanogen Pharmaceutical Biotechnology), and inactivated vaccine of ERUCOV-VAC (Erciyes University, Turkey), are other candidate vaccines under phase 3 trials. Moreover, two DNA-based vaccines INO-4800 (Inovio Pharmaceuticals) and AG0301-COVID19 (AnGes/Takara Bio/Osaka University), three protein subunit vaccines SCB-2019 + AS03 (Clover Biopharmaceuticals Inc./GSK/Dynavax),UB-612 (COVAXX/United Biomedical Inc), and MF59 adjuvanted SARS-CoV-2 Sclamp vaccine (CSL Ltd. + Seqirus + the University of Queensland), GRAd-COV2 vaccine based on replication-defective Simian Adenovirus (GRAd) encoding S (ReiThera + Leukocare + Univercells), coronavirus-like particle COVID-19 vaccine (Medicago Inc), COVID-19 inactivated vaccine (Shifa Pharmed Industrial Co), and mRNA-based vaccine mRNA-1273.211 (ModernaTX, Inc.) are currently in combining phases 2 and 3 trials. Vaccine candidates are listed in Table 3.

**Table 3:** List of COVID-19 vaccine candidates

Type of candidate vaccine	Vaccine platform	Company/University	Vaccine characteristics/immunogen
ARCT-154	RNA	Arcturus Therapeutics	Self-replicating mRNA coding spike protein, encapsulated in lipid nanoparticles
Inactivated SARS-CoV-2 vaccine (Vero cell)	Inactivated virus	Beijing Minhai Biotechnology Co	Inactivated SARS-CoV-2
WIBP COVID-19 vaccine	Inactivated virus	Sinopharm; China National Biotec Group Co; Wuhan Institute of Biological Products	Inactivated SARS-CoV-2
Inactivated SARS-CoV-2 vaccine (Vero cell), vaccine name BBIBP-CorV	Inactivated virus	Sinopharm; China National Biotec Group Co; Wuhan Institute of Biological Products, Beijing Institute of Biological Products	Inactivated SARS-CoV-2
SARS-CoV-2 rS/Matrix M1-Adjuvant	Protein subunit	Novavax	Full-length recombinant SARS CoV-2
BNT162b2 (3 LNP-mRNAs), also known as "Comirnaty"	RNA	Pfizer/BioNTech + Fosun Pharma	RNA based vaccine
Recombinant SARS-CoV-2 vaccine (CHO Cell) Zifivax (ZF2001)	Protein subunit	Anhui Zhifei Longcom Biopharmaceutical + Institute of Microbiology, Chinese Academy of Sciences Zhongyianke Biotech	Recombinant spike protein
CVnCoV Vaccine	RNA	CureVac AG	RNA based vaccine
SARS-CoV-2 vaccine (vero cells)	Inactivated virus	Institute of Medical Biology + Chinese Academy of Medical Sciences	Inactivated SARS-CoV-2
MVC-COV1901	Protein subunit	Medigen Vaccine Biologics + Dynavax + National Institute of Allergy and Infectious Diseases (NIAID)	Spike-2P protein + adjuvant CpG 1018
CIGB-66	Protein subunit	Center for Genetic Engineering and Biotechnology (CIGB)	RBD + aluminium hydroxide
Recombinant protein vaccine S-268019	Protein subunit	Shionogi	using Baculovirus expression vector system
TURKOVAC	Inactivated virus	Erciyes University and the Health Institutes of Turkey (TUSEB)	Inactivated SARS-CoV-2
Recombinant SARS-CoV-2 Fusion Protein Vaccine (V-01)	Protein subunit	Livzon Pharmaceutical	Recombinant spike protein
Noora Vaccine	Protein subunit	Bagheiat-allah University of Medical Sciences/AmitisGen	RBD protein recombinant SARS-CoV-2 vaccine
COVID-19 Vaccine Hipra	Protein subunit	Laboratorios Hipra, S.A	Recombinant protein RBD fusion dimer adjuvanted vaccine
SCTV01C	Protein subunit	Sinocelltech Ltd	A Bivalent Recombinant Trimeric S Protein vaccine against SARS-CoV-2 Variants
CoviVac	Inactivated virus	Chumakov Federal Scientific Center for Research and Development of Immune-and-Biological Products	Inactivated Whole Virion Concentrated Purified Vaccine
Omicron COVID-19 inactivated Vaccine (Vero Cell)	Inactivated virus	China National Biotec Group Company Limited	Inactivated SARS-CoV-2
MV C-COV1901	Protein subunit	Medigen Vaccine Biologics/Dynavax/NIAID	Spike protein with aluminum and CpG 1018 as adjuvant
DelNS1-2019-nCoV-RBD-OPT1	Viral vector (Replicating)	University of Hong Kong, Xiamen University and Beijing Wantai Biological Pharmacy	Intranasal flu-based-RBD
COVAX-19	Protein subunit	Vaxine Pty Ltd	Recombinant spike protein with Advax™ adjuvant
Razi Cov Pars	Protein subunit	Razi Vaccine and Serum Research Institute	Recombinant spike protein
Recombinant SARS-CoV-2 Fusion Protein Vaccine (V-01)	Protein subunit	Guangdong Provincial Center for Disease Control and Prevention/Gaozhou Center for Disease Control and Prevention	Recombinant spike protein
GRAd-COV2	Viral vector (Non-replicating)	ReiThera/Leukocare/Univercells	Replication-defective Gorilla Adenovirus vector-based vaccine, which codes spike protein
VXA-CoV2-1	Viral vector (Non-replicating)	Vaxart	Ad5 vector-based vaccine that expresses a SARS-CoV-2 antigen and a dsRNA adjuvant to increase immunogenicity, which given orally
COVI-VAC	Live attenuated virus	Codagenix/Serum Institute of India	Live attenuated SARS-CoV-2
ChulaCov19	RNA	Chulalongkorn University	mRNA vaccine
BBV154	Viral vector (Non-replicating)	Bharat Biotech International Limited	Vaccine based on Adenoviral vector
PTX-COVID19-B	RNA	Providence Therapeutics	Vaccine based on mRNA
SC-Ad6-1	Viral vector (Non-replicating)	Tetherex Pharmaceuticals Corporation	Adneviral vector vaccine
ReCOV	Protein subunit	Jiangsu Rec-Biotechnology	Recombinant two-component S and RBD protein COVID-19 vaccine (CHO cell)
RBD protein recombinant SARS-CoV-2 vaccine	Protein subunit	Bagheiat-allah University of Medical Sciences	Recombinant RBD of the spike protein of SARS-CoV-2
ABNCoV2	Virus like particle	Radboud University	ABNCoV2 capsid VLP with or without adjuvant MF59
GX-19N	DNA	Genexine Consortium	Vaccine expressing spike protein antigen
EpiVacCorona	Protein subunit	Federal Budgetary Research Institution State Research Center of Virology and Biotechnology "Vector"	Peptide antigens of proteins of SARS-CoV-2, conjugated to a carrier protein and adsorbed on aluminum hydroxide as adjuvant
Dendritic cell vaccine AV-COVID-19	Protein subunit	Center for Genetic Engineering and Biotechnology (CIGB)	A vaccine containing autologous dendritic cells loaded with SARS-CoV-2 antigens, with or without GM-CSF
rVSV-SARS-CoV-2-S Vaccine	Protein subunit	Center for Genetic Engineering and Biotechnology (CIGB)	Replication-competent SARS-CoV-2 spike protein
BECOV2	Protein subunit	Biological ELimited	Protein subunit vaccine
GBP510	Protein subunit	SK Bioscience Co., Ltd	A recombinant surface protein vaccine with adjuvant AS03 (aluminum hydroxide)
COVAC-1 and COVAC-2 sub-unit vaccine	Protein subunit	University of Saskatchewan	(spike protein) + SWE adjuvant
SARS-CoV-2-RBD-Fc fusion protein	Protein subunit	University Medical Center Groningen/Akston Biosciences Inc	Recombinant RBD with Fc fusion protein
DS-5670a	RNA	Daiichi Sankyo Co., Ltd	mRNA vaccine
Recombinant SARS-CoV-2 Vaccine (CHO cell)	Protein subunit	National Vaccine and Serum Institute, China	Subunit vaccine

## Conclusions

As COVID-19 is a new disease, efforts are being continued to find a suitable treatment for it. To date, the only approved drug with significant efficacy in the clinical cure of patients with COVID-19 is Paxlovid. Therapeutic agents to treat COVID-19 are selected because of their previously documented antiviral activity against MERS and SARS or other virus infections. There is an immediate requirement to obtain a drug or vaccine with an approved efficacy for the treatment or prevention of COVID-19. After the recent announcement of the efficacy of several COVID-19 vaccine candidates in the protection of disease, a comprehensive strategy is now needed to make sure vaccination of the global population in the next steps. Although efficient vaccination and preventative prevention attempts can be crucial, it is not yet clear whether these vaccines can end the COVID-19 pandemic.

## Data Availability

The datasets used and/or analyzed during the current study are available from the corresponding author upon reasonable request.
